# S-Adenosylmethionine Decreases Bacterial Translocation, Proinflammatory Cytokines, Oxidative Stress and Apoptosis Markers in Hepatic Ischemia-Reperfusion Injury in Wistar Rats

**DOI:** 10.3390/antiox12081539

**Published:** 2023-07-31

**Authors:** Sergio Valdés, Sergio D. Paredes, Carmen García Carreras, Pilar Zuluaga, Lisa Rancan, Beatriz Linillos-Pradillo, Javier Arias-Díaz, Elena Vara

**Affiliations:** 1 Department of Biochemistry and Molecular Biology, School of Medicine, Complutense University of Madrid, Avda. Complutense, s/n, 28040 Madrid, Spain; svaldes@ucm.es (S.V.); cgc2910@hotmail.com (C.G.C.); lisaranc@ucm.es (L.R.); beatlini@ucm.es (B.L.-P.); evaraami@ucm.es (E.V.); 2 Department of Physiology, School of Medicine, Complutense University of Madrid, Avda. Complutense, s/n, 28040 Madrid, Spain; 3 Departmental Unit of Biostatistics—Department of Statistics and Operations Research, School of Medicine, Complutense University of Madrid, Avda. Complutense, s/n, 28040 Madrid, Spain; pilarzul@med.ucm.es; 4 Department of Surgery, School of Medicine, Complutense University of Madrid, Avda. Complutense, s/n, 28040 Madrid, Spain; javierar@ucm.es

**Keywords:** S-adenosylmethionine, ischemia-reperfusion, liver, cytokine, endotoxin, lipid hydroperoxide, phosphatidylcholine, caspase, vena cava, portal vein

## Abstract

Hepatic ischemia/reperfusion injury (IRI) can seriously impair liver function. It is initiated by oxidative stress, resulting in inflammation and apoptosis-induced cellular damage. Glutathione (GSH) prevents oxidative stress. S-Adenosylmethionine (SAMet) is a GSH synthesis precursor that avoids the deficit in SAMet-synthetase activity and contributes to intracellular ATP repletion. It also acts as a methyl group donor, stabilizing hepatocyte membranes, among other functions. This study investigated the effect of SAMet on bacterial translocation and levels of proinflammatory cytokines, oxidative stress and apoptosis markers in male Wistar rats subjected to hepatic IRI. Animals were randomly divided into six groups: (1) sham operation, (3) animals undergoing 60 min of ischemia of the right lateral lobe for temporary occlusion of the portal vein and hepatic artery plus 10 min of reperfusion, and (5) the same as (3) but with a reperfusion period of 120 min. Groups 2, 4 and 6, respectively, are the same as (1), (3) and (5), except that animals received SAMet (20 mg/kg) 15 min before ischemia. GSH, ATP, lipid peroxidation (LPO), TNF-α, IL-1β, IL-6, total caspase-1 and caspase-9, total and cleaved caspase-3, and phosphatidylcholine were determined in the liver. Endotoxin, TNF-α, IL-1β, IL-6, IL-10 and LPO in vena cava and portal vein blood samples were also measured. Endotoxin and LPO levels as well as proinflammatory cytokines and apoptotic markers increased significantly in animals undergoing IRI, both after 10 and 120 min of reperfusion. IRI produced a significant decrease in GSH, ATP, portal IL-10 and phosphatidylcholine. SAMet treatment prevented these effects significantly and increased survival rate. The study suggests that SAMet exerts protective effects in hepatic IRI.

## 1. Introduction

Situations of hepatic ischemia-reperfusion in clinical practice are frequent, since they occur not only in liver transplantation, but also in various surgical interventions on the liver in which temporary clamping of the hepatic pedicle (Pringle maneuver) is required to reduce blood loss. In addition, ischemia-reperfusion microphenomena are becoming increasingly relevant in the pathophysiology of various states of shock, especially septic shock.

The mechanisms of damage or injury due to ischemia and subsequent reperfusion (ischemia-reperfusion injury or IRI) are extensive and varied including: (1) ATP depletion during ischemia, and its subsequent degradation to hypoxanthine, leading to a superoxide radical at reperfusion [1]; (2) the loss of function of calcium transporters and the marked intracellular increase in this ion which produces the activation of proteases that degrade the cytoskeleton with consequent cell deformation (blebbing) as well as the activation of phospholipase A2, which produces an increase in arachidonic acid derivatives, and the release of cytochrome c with the activation of caspases, which can lead to cell death by apoptosis [2]; (3) damage to the lipids that make up the cell membrane (lipid peroxidation; LPO), through the oxidation of polyunsaturated fatty acids, with a high production of alkyl and peroxyl radicals; and (4) the destabilization of various nuclear transcription factors and increased vascular permeability [3].

Glutathione (GSH) is the key to the cellular protection mechanism against free-radical-mediated toxicity, constituting a natural defense mechanism [4]. S-Adenosylmethionine (SAMet) is an intracellular amino acid and enzyme co-factor that acts in multiple transmethylation reactions as a methyl donor, providing methyl groups to different substrates: DNA, proteins, lipids, including phosphatidylcholine, or RNA [5,6]. The endogenous biosynthesis of SAMet takes place through the action of SAMet-synthetase. GSH depletion can enhance toxicity due to oxygen free radicals. Since one of the main actions of SAMet is to maintain elevated GSH levels [7], this could be a very important protective mechanism against the harmful effects induced by oxidative-stress-inducing events.

A reduction of GSH in the liver, as occurs in patients with liver damage, seems to initiate a vicious circle, since such a depletion may lead to the inactivation of SAMet-synthetase, resulting in a further decrease in GSH levels, with a consequent worsening in the function of this enzyme [8]. Moreover, during oxidative stress, sulfur amino acid homeostasis is compromised and GSH levels, a key antioxidant in the body, are lowered [9]. Given that oxygen radicals are involved in liver damage secondary to ischemia-reperfusion, as well as the fact that SAMet is capable of increasing the GSH content of hepatocytes, among other possible protective mechanisms, and as part of the modern approach of the early antagonization of specific mediators of cell damage that ultimately lead to organ failure, we hypothesized here that this molecule may be beneficial in an experimental model of hepatic IRI.

Thus, the aim of the present work was to evaluate the effect of SAMet on inflammation, oxidative stress and apoptosis markers (cytokines, LPO and caspases) as well as GSH, ATP, endotoxin and phosphatidylcholine levels and survival rate in male Wistar rats subjected to hepatic IRI.

## 2. Materials and Methods

### 2.1. Animals

Male Wistar rats weighing 250–300 g were housed in a facility subjected to automatic control of light–dark cycles (12 h of light, from 08:00 to 20:00, and 12 h of darkness) and temperature (22 ± 2 °C) and fed with a standard diet supplied by Panlab (Barcelona, Spain). The animals were treated humanely, and the Ethical Norms in Animal Research dictated by the European Union were always strictly followed (2010/63/UE).

### 2.2. Experimental Design

After the rats were kept in a fasting situation for 24 h, they were divided into two subgroups: some animals were subjected to the surgical procedure of IRI and left to live, with free access to food and water for the survival study; others were randomly distributed into six groups and sacrificed after the operation (groups 1 and 2), or at 10 min (groups 3 and 4) or 120 min (groups 5 and 6) after reperfusion, and blood samples from the vena cava and portal vein and liver were taken:

Group 1: Underwent a sham operation, being used as a basal group.

Group 2: The same as group 1, except that SAMet was administered to the animals, at a dose of 20 mg/kg/b.w., 15 min before the operation.

Group 3: Underwent 60 min of ischemia of the right lateral lobe by temporary occlusion of the portal vein and hepatic artery (its corresponding branches), according to the model described by Hayashi et al. [10]. At the time of right lobe completing 10 min of reperfusion, the middle and left lateral lobes (i.e., approximately 80% of the hepatic mass) were removed.

Group 4: The same as group 3, except that SAMet was administered to the animals, at a dose of 20 mg/kg/b.w., 15 min before the operation.

Group 5: The same as group 3, but reperfusion lasted 120 min.

Group 6: The same as group 5, except that SAMet was administered to the animals, at a dose of 20 mg/kg/b.w., 15 min before the operation.

### 2.3. Sample Collection

Rats were anesthetized with diethyl ether by inhalation. Then, they were injected with the corresponding product (SAMet at a dose of 20 mg/kg/b.w., diluted in 0.2 mL of saline solution or an equivalent volume of saline solution alone), slowly, to avoid an abrupt overload of the animal’s cardiocirculatory system. Systemic heparinization (at a dose of 100 IU/kg/b.w. of sodium heparin) was also performed. These injections were carried out in the internal jugular vein by percutaneous puncture.

Animals were approached by bilateral subcostal laparotomy and underwent the corresponding operation. Both liver and blood samples were collected from the same animals. Liver samples were obtained from the right hepatic lobe. Blood samples were collected from the vena cava and portal vein. Specifically, anesthetized rats were placed in a supine position on a clean surgical surface, ensuring that the rat’s limbs were adequately secured to prevent sudden movements during the procedure. Surgical sites were shaven and scrubbed with 2% solution of chlorhexidine gluconate. Using a clean surgical technique, a midline abdominal incision was made, taking care to avoid injuring any vital structures.

The liver was covered with sterile saline-soaked gauze, retracted cephalad and secured with curved miniature retractors. Other viscera overlying the portal vein were retracted to the animal’s left. All exposed viscera were kept covered with sterile saline-soaked gauze.

With the aid of an illuminated magnifying glass, an area of approximately 1.5 cm in diameter was exposed over the portal vein. The bile duct was freed to the main portal branches and retracted to the rat’s left. The hepatic artery was bluntly dissected from the left side of the portal vein behind the gastroduodenal vein. The proper hepatic artery was freed up from the portal vein to the lobular branches.

The portal vein was temporarily clamped caudad to the hepatic lobular branches with a microvascular clip. The wall of the portal vein was picked up with fine-tipped forceps and pierced with a 24 ga neonatal cannula/needle unit angled toward the liver. The cannula was pushed off the needle and advanced about 3 mm and locked with a 5 mL syringe. After 3 mL of portal blood was sampled, the cannula was removed and gentle pressure using a cotton-tipped swab was applied to the portal vein puncture site to achieve hemostasis.

Afterwards, abdominal viscera were retracted cephalad to expose the inferior vena cava, which was isolated from surrounding tissues using microsurgical scissors and forceps, exercising caution to avoid any damage to the vessel. The anterior vein wall was picked up with fine-tipped forceps and pierced with another 24 ga cannula/needle unit angled cephalad. Once advanced 3 mm into the vein, the cannula was attached to a 10 mL syringe and blood sampling was carried out until complete exsanguination of the rat.

Subsequently, blood samples were centrifuged to separate the plasma and stored frozen, together with the tissue samples, until the determination of the biomarkers under investigation.

### 2.4. Glutathione (GSH)

A specific photometric micromethod [11], based on the non-enzymatic reaction of GSH with 5-5′-dithiobis-(2-nitrobenzoic acid) (DTNB), was used for its determination in liver tissue, leading to the formation of 2-nitro-5-tiobenzoic acid (TNB) and GSSG. The rate of formation of TNB was followed spectrophotometrically at 412 nm.

### 2.5. ATP

It was determined in liver tissue using a specific kit (Sigma, St. Louis, MO, USA), based on the following reactions: (1) The enzyme phosphoglycerate phosphokinase (PGK) catalyzes the reaction: ATP + 3-phosphoglycerate = ADP + 1,3-bisphosphoglycerate; (2) The enzyme glyceraldehyde phosphate dehydrogenase (GAPD) is involved in the following reaction: 1,3-bisphosphoglycerate + NADH + H^+^ = Glyceraldehyde-3-P + NAD^+^ + phosphate. The decrease in absorbance at 340 nm, due to the oxidation of NADH to NAD^+^, was proportional to the increase in ATP in the medium.

### 2.6. Endotoxin

A specific colorimetric kit for a bacterial endotoxin was used (Sigma, St. Louis, MO, USA) for its determination in plasma of the vena cava and portal vein. It was based on the fact that Gram (-) bacterial endotoxin catalyzes the activation of a proenzyme present in Limulus Amebocyte Lysate (LAL). The initial rate of activation depended on the concentration of endotoxin present.

### 2.7. Lipid Peroxidation (LPO)

Plasma lipid hydroperoxide levels were determined using a specific kit (Kamiya Biomedical Company, Seattle, WA, USA). Their rationale is based on the reaction of hydroperoxides with a derivative of methylene blue, 10-N-methylcarbamoyl-3,7-dimethylamino-10-H-phenothiazine (MCDP), in a reaction catalyzed by hemoglobin, to give rise to the formation of methylene blue. LPO was quantified colorimetrically by measuring the methylene blue formed. Quantification of LPO in liver homogenate was carried out by means of a lipid hydroperoxide assay kit according to the manufacturer’s instructions (Cayman Chemical, Ann Arbor, MI, USA). It measures the hydroperoxides directly utilizing the redox reactions with ferrous ions. The amount of lipid hydroperoxides was obtained from the linear regression of the standard curve substituting corrected absorbance values for each sample. This procedure eliminates any interference caused by hydrogen peroxide or endogenous ferric ions in the sample and provides a more sensitive and reliable assay for LPO.

### 2.8. Cytokines TNF-α, IL-1β, IL-6 and IL-10

They were determined in liver and plasma samples by ELISA using specific commercial kits (bioNova científica S.L., Madrid, Spain).

### 2.9. Phosphatidylcholine

It was determined by the incorporation of labeled choline (^3^H-choline) into the lipid fraction. For this purpose, the rats were injected with the labeled isotope 2 h before obtaining the samples. For the extraction of phosphatidylcholine from the liver tissue samples, a sample of the liver tissue was homogenized in chloroform/methanol (2:1) to a final dilution of 1/17. The sample was then equilibrated at room temperature for 1 h and filtered into a ground-glass tube with a ground-glass stopper. The crude extract was mixed with 0.2 times its volume of Folch solution, and the two phases of the mixture were separated by centrifugation. After aspirating the upper phase, three washes were performed with UPS (50% of the original volume), centrifuging after each wash, and the upper phase was discarded. Finally, the lower phase and the remnant of the upper phase were converted to one phase by the addition of methanol, and the resulting solution was evaporated under vacuum. The dried extract was redissolved with chloroform/methanol and a 30 µL sample was applied to chromatography plates (Silica Gel 60, 20 × 20) previously activated for 1 h at 110 °C. The phosphatidylcholine fraction was separated using chromatography in two runs using two mixtures of solvents.

### 2.10. Total Caspase-1, -3 and -9 and Cleaved Caspase-3

They were determined in liver tissue by ELISA using specific commercial kits (Abcam, Waltham, MA, USA).

### 2.11. Survival Study

After the surgical procedure, and SAMet treatment where appropriate, rats were randomly chosen from each experimental group and allowed to live, with free access to food and water, for a survival study. Animals from groups (3) and (5) were assigned to the group IRI, while those belonging to the group IRI + SAMet were from groups (4) and (6). All animals that survived more than 72 h after the surgical procedure were considered permanent survivors.

### 2.12. Statistical Analysis

The description of quantitative variables was made by means of mean and standard deviation. Comparison of the survival percentage was made using a chi-square test. For the comparison between groups of quantitative variables, given that the sample sizes of the groups are equal (n = 10), equality of variances was previously contrasted using Levene’s test, and then, based on the results of this test, the means were compared using ANOVA (with Tukey’s post-hoc comparisons) or Welch’s generalization (with Games–Howell post-hoc comparisons). The significance level of 5% was considered for the tests. All data were analyzed using SPSS v.27 and Excel.

## 3. Results

### 3.1. Effect of IRI and SAMet Treatment on ATP and GSH Levels in Liver

**Figure 1:**
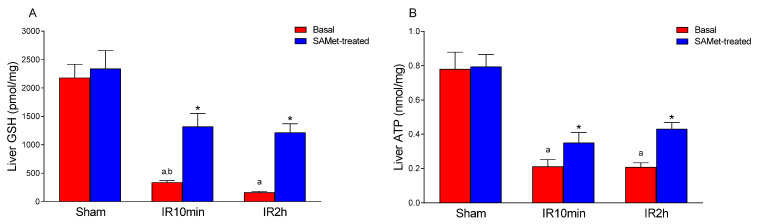
The figure shows the mean levels of GSH (pmol/mg) (**A**) and ATP (nmol/mg) (**B**) in liver tissue from male Wistar rats that were treated 15 min before surgery with 20 mg/kg SAMet diluted in 0.2 mL of saline in the groups thus indicated (sham + SAMet, IR10min + SAMet and IR2h + SAMet; blue columns) or 0.2 mL of saline exclusively in the basal groups (sham, IR10min and IR2h; red columns). (a) vs. sham, (b) vs. IR2h, and (*) vs. their respective non-treated group.

As for GSH and ATP in liver tissue (Figure 1), a comparison of the means of the six groups was significant in both determinations (*p* < 0.001). The sham procedure itself did not inherently reduce the levels of either vs. the corresponding SAMet-treated sham group (*p* = 0.999), but IRI did. We observed that SAMet treatment markedly enhanced the decrease in GSH (Figure 1A) and ATP (Figure 1B), both at 10 min (IR10min; GSH 339.923 ± 28.4623 pmol/mg tissue vs. 1320.531 ± 230.3826 pmol/mg tissue, *p* < 0.001; and ATP 0.21 ± 0.04 nmol/mg tissue vs. 0.35 ± 0.06 nmol/mg tissue, *p* < 0.001) and at 120 min (IR2h; GSH 160.884 ± 18.36 pmol/mg tissue vs. 1215.337 ± 153.78 pmol/mg tissue, *p* < 0.001; and ATP 0.209 ± 0.025 nmol/mg tissue vs. 0.4309 ± 0.038 nmol/mg tissue, *p* < 0.001) of reperfusion.

### 3.2. Effect of IRI and SAMet Treatment on Endotoxin in Cava and Portal Veins

**Figure 2:**
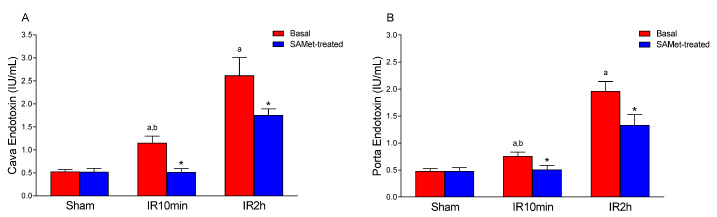
The figure shows the mean levels of endotoxin (IU/mL) in the vena cava (**A**) and portal vein (**B**). Samples were taken from vena cava and portal vein blood of male Wistar rats treated 15 min before surgery with 20 mg/kg SAMet diluted in 0.2 mL of saline in the groups thus indicated (sham + SAMet, IR10min + SAMet and IR2h + SAMet; blue columns) or 0.2 mL of saline exclusively in the basal groups (sham, IR10min and IR2h; red columns). (a) vs. sham, (b) vs. IR2h, and (*) vs. their respective non-treated group.

Endotoxin levels (Figure 2A,B), reflecting bacterial translocation, were substantially increased in rats subjected to IRI, but less so in those treated with SAMet. While there was no difference between the non-treated and SAMet-treated sham groups (*p* = 0.999), higher levels were observed in the non-treated groups after 10 min of reperfusion (IR10min) compared with the lower levels observed in those treated with SAMet in the vena cava (Figure 2A) (1.149 ± 0.15 IU/mL plasma vs. 0.513 ± 0.079 IU/mL plasma, *p* < 0.001) and in the portal vein (Figure 2B) (0.758 ± 0.073 IU/mL plasma vs. 0.508 ± 0.079 IU/mL plasma, *p* < 0.001). After 120 min of reperfusion (IR2h), lower levels were also observed in the SAMet-treated groups compared to non-treated animals in both the vena cava (2.618 ± 0.387 IU/mL plasma vs. 1.757 ± 0.133 IU/mL plasma, *p* < 0.001) and portal vein (1.964 ± 0.176 IU/mL plasma vs. 1.333 ± 0.194 IU/mL plasma, *p* < 0.001).

### 3.3. Effect of IRI and SAMet Treatment on LPO Levels in Liver and Cava and Portal Veins

**Figure 3:**
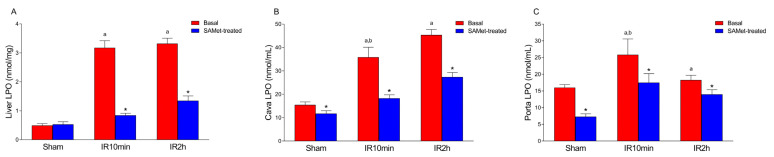
The figure shows the mean levels of LPO in the liver (nmol/mg) (**A**) and in the vena cava and portal vein (nmol/mL) (**B**,**C**). Samples were taken, as appropriate, from the liver tissue, vena cava blood or portal vein blood of male Wistar rats treated 15 min before surgery with 20 mg/kg SAMet diluted in 0.2 mL of saline in the groups thus indicated (sham + SAMet, IR10min + SAMet and IR2h + SAMet; blue columns) or 0.2 mL of saline exclusively in the basal groups (sham, IR10min and IR2h; red columns). (a) vs. sham, (b) vs. IR2h, and (*) vs. their respective non-treated group.

The comparison of the LPO mean levels among the six groups was significant in liver (*p* < 0.001; Figure 3A) as well as in both cava (*p* < 0.001) and portal (*p* < 0.001) veins (Figure 3B,C).

The mean LPO in the liver was not significantly different between the non-treated and SAMet-treated sham groups (*p* = 0.994). IRI increased liver LPO levels significantly at both 10 min and 120 min of reperfusion (*p* < 0.001). SAMet treatment significantly decreased mean liver LPO levels at 10 min of reperfusion (IR10min vs. IR10min + SAMet groups; *p* < 0.001). Likewise, SAMet treatment significantly decreased mean liver LPO levels at 120 min of reperfusion (IR2h vs. IR2h + SAMet groups; *p* < 0.001).

The mean LPO was significantly lower in the SAMet-treated sham group than in the non-treated sham group in both the vena cava (*p* = 0.006) and portal vein (*p* < 0.001). SAMet treatment significantly decreased mean LPO levels at 10 min of reperfusion (IR10min vs. IR10min + SAMet groups) and at 120 min of reperfusion (IR2h vs. IR2h + SAMet groups) in both veins (*p* < 0.001).

### 3.4. Effect of IRI and SAMet Treatment on Cytokine Levels (TNF-α, IL-1β, IL-6 and IL-10) in Liver and Cava and Portal Veins

**Figure 4:**
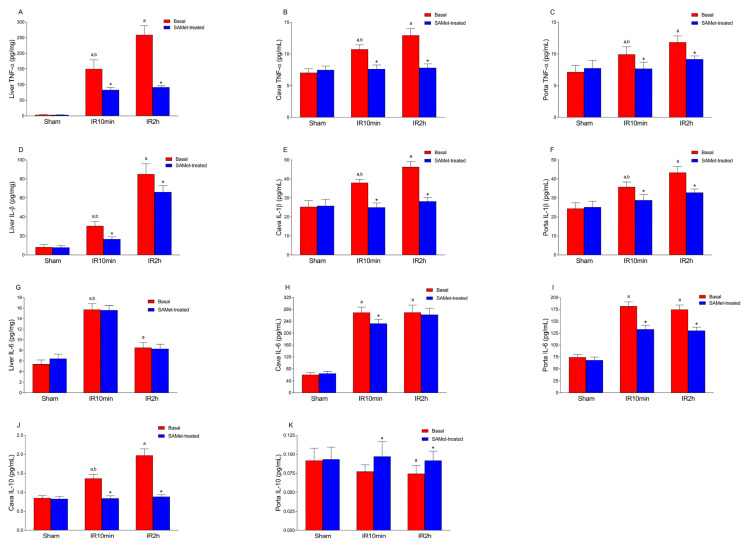
The figures show the mean levels of TNF-α, IL-β and IL-6 in the liver (pg/mg) (**A**,**D**,**G**) and the mean levels of TNF-α, IL-β, IL-6 and IL-10 in the vena cava (pg/mL) (**B**,**E**,**H**,**J**) and portal vein (pg/mL) (**C**,**F**,**I**,**K**). Samples were taken, as appropriate, from the liver tissue, vena cava blood or portal vein blood of male Wistar rats treated 15 min before surgery with 20 mg/kg SAMet diluted in 0.2 mL of saline in the groups thus indicated (sham + SAMet, IR10min + SAMet and IR2h + SAMet; blue columns) or 0.2 mL of saline exclusively in the basal groups (sham, IR10min and IR2h; red columns). (a) vs. sham, (b) vs. IR2h, and (*) vs. their respective non-treated group.

With respect to TNF-α (Figure 4A) and IL-1β (Figure 4D), both measured in liver tissue, these cytokines presented very low levels under normal conditions with no significant difference between the non-treated and SAMet-treated sham groups (*p* = 0.999), but increased markedly at 10 min after reperfusion in rats subjected to IRI, and this increase was reduced with SAMet (TNF-α 150.148 ± 29.24 pg/mg tissue vs. 82.899 ± 8.003 pg/mg tissue, *p* < 0.001; IL-1β 30.58 ± 4.64 pg/mg tissue vs. 16.69 ± 2.44 pg/mg tissue, *p* < 0.001). Additional measurements that were taken in the groups after 120 min of reperfusion also demonstrated a significant reduction in SAMet-treated animals when compared to non-treated animals (TNF-α 259.028 ± 29.42 pg/mg tissue vs. 91.64 ± 4.74 pg/mg tissue, *p* < 0.001; IL-1β 85.09 ± 10.85 pg/mg tissue vs. 66.19 ± 6.89 pg/mg tissue, *p* < 0.001). Similar effects were also observed in cava and portal veins in both TNF-α (Figure 4B,C) and IL-1β (Figure 4E,F).

IL-6 levels also increased with IRI (Figure 4G–I). However, levels in liver tissue after IRI did not decrease with SAMet (Figure 4G). Thus, in liver tissue, the difference between the non-treated and SAMet-treated sham groups was not significant (*p* = 0.442), nor was it significant at 10 min (IR10min; *p* = 0.898) or 120 min (IR2h; *p* = 0.858) after reperfusion.

As for circulating IL-6, the mean was not significantly different between the non-treated and SAMet-treated sham groups in both cava (Figure 4H) (*p* = 0.736) and portal (Figure 4I) (*p* = 0.585) veins. SAMet treatment decreased IL-6 levels after 10 min of reperfusion in the cava vein (Figure 4H) (269.68 ± 18.12 pg/mL plasma vs. 232.63 ± 14.315 pg/mL plasma, *p* < 0.001) as well as in the portal vein (Figure 4I) (182.015 ± 8.76 pg/mL plasma vs. 133.24 ± 8.24 pg/mL plasma, *p* < 0.001). At 120 min of reperfusion, levels also decreased significantly in the portal vein (174.69 ± 9.46 pg/mL plasma vs. 130.297 ± 7.45 pg/mL plasma, *p* < 0.001) but not in the cava vein (*p* = 0.984).

IL-10 measured in the vena cava (Figure 4J) and portal vein (Figure 4K) showed no significant variation (*p* = 0.999) between the non-treated and SAMet-treated sham groups. However, in SAMet groups, its levels in the portal vein increased both at 10 min (0.077 ± 0.009 pg/mL plasma vs. 0.097 ± 0.019 pg/mL plasma, *p* < 0.001) and 120 min (0.0746 ± 0.01 pg/mL plasma vs. 0.0918 ± 0.0125 pg/mL plasma, *p* < 0.001) after reperfusion (IR10min and IR2h, respectively; Figure 4K). For the vena cava, IL-10 decreased in the SAMet group both at 10 min (IR10min; 1.365 ± 0.102 pg/mL plasma vs. 0.837 ± 0.07 pg/mL plasma, *p* < 0.001) and 120 min (IR2h; 1.97 ± 0.171 pg/mL plasma vs. 0.884 ± 0.052 pg/mL plasma, *p* < 0.001) after reperfusion (Figure 4J).

### 3.5. Effect of IRI and SAMet Treatment on Phosphatidylcholine in Liver

**Figure 5:**
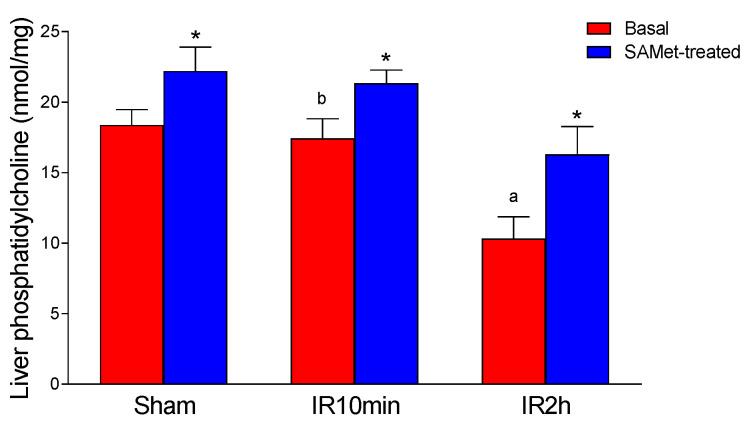
The figure shows the mean levels of phosphatidylcholine (nmol/mg) in liver tissue from male Wistar rats that were treated 15 min before surgery with 20 mg/kg SAMet diluted in 0.2 mL of saline in the groups thus indicated (sham + SAMet, IR10min + SAMet and IR2h + SAMet; blue columns) or 0.2 mL of saline exclusively in the basal groups (sham, IR10min and IR2h; red columns). (a) vs. sham, (b) vs. IR2h, and (*) vs. their respective non-treated group.

Phosphatidylcholine was significantly higher in the SAMet-treated sham group than in the non-treated sham group in liver tissue (*p* < 0.001) (Figure 5). IRI significantly decreased the phosphatidylcholine levels after a reperfusion of 120 min (*p* < 0.001), but no significant changes were found with respect to the non-treated sham group after a reperfusion of 10 min. SAMet treatment significantly increased mean phosphatidylcholine levels at both 10 min (IR10min vs. IR10min + SAMet groups) and 120 min of reperfusion (IR2h vs. IR2h + SAMet groups) (*p* < 0.001).

### 3.6. Effect of IRI and SAMet Treatment on Total Caspase-1, -3, and -9 and Cleaved Caspase-3 in Liver

**Figure 6:**
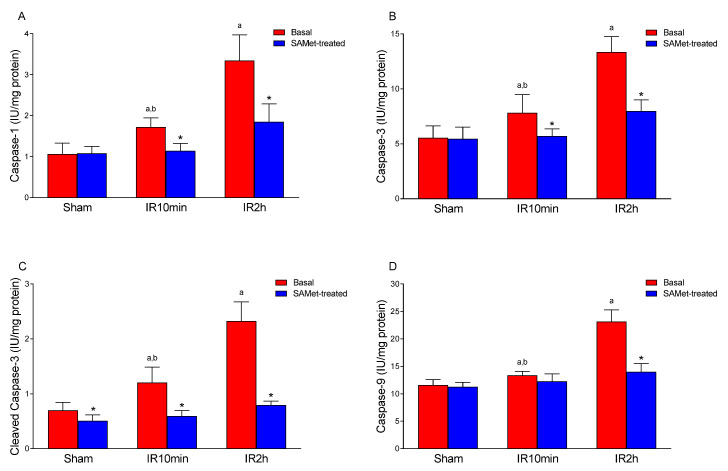
The figure shows the mean levels of total caspase-1, -3 and -9 (IU/mg protein) ((**A**,**B**,**D**), respectively) and cleaved caspase-3 (**C**) in liver tissue from male Wistar rats that were treated 15 min before surgery with 20 mg/kg SAMet diluted in 0.2 mL of saline in the groups thus indicated (sham + SAMet, IR10min + SAMet and IR2h + SAMet; blue columns) or 0.2 mL of saline exclusively in the basal groups (sham, IR10min and IR2h; red columns). (a) vs. sham, (b) vs. IR2h, and (*) vs. their respective non-treated group.

No significant differences were found in liver tissue total caspase levels when comparing the non-treated and SAMet-treated sham groups (Figure 6). IRI increased the liver caspase-1 (Figure 6A), -3 (Figure 6B) and -9 (Figure 6D) values significantly (*p* < 0.001) and SAMet treatment generally counteracted this effect at both 10 and 120 min of reperfusion in a significant manner (*p* < 0.001), with the exception of caspase-9 after a reperfusion of 10 min, where the decrease was not significant with respect to the non-treated animals subjected to the surgery. Regarding cleaved caspase-3, the mean was significantly lower in the sham group treated with SAMet than in the non-treated sham group (*p* = 0.049). SAMet treatment significantly decreased mean cleaved caspase-3 levels at 10 min of reperfusion (IR10min vs. IR10min + SAMet groups; *p* < 0.001). The same effect was observed at 120 min of reperfusion (IR2h vs. IR2h + SAMet groups; *p* < 0.001).

### 3.7. Effect of IRI and SAMet Treatment on Survival Rate

**Figure 7:**
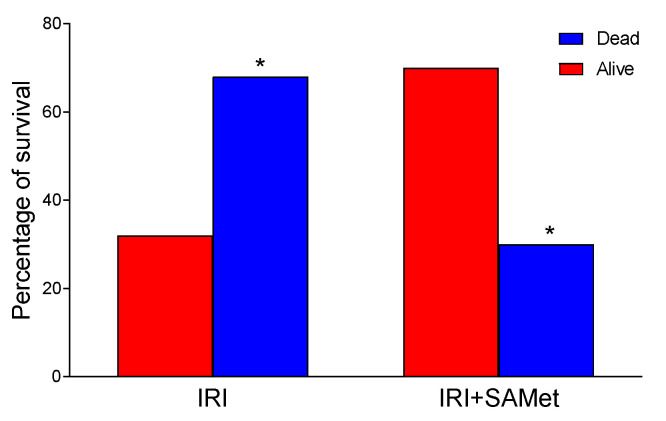
The figure shows the percentage of survival in male Wistar rats that were treated 15 min before surgery with 20 mg/kg SAMet diluted in 0.2 mL of saline (IRI + SAMet) or 0.2 mL of saline exclusively (IRI). Red columns represent animals that survived more than 72 h after the surgery. Blue columns represent animals that lived less than 72 h after the surgery. (*) vs. their respective group of surviving animals.

Mortality in the group subjected to IRI reached 68%, decreasing significantly to 30% in rats previously treated with SAMet (*p* < 0.01) (Figure 7). The survival rate in the IRI group was 32%. However, in the group that received SAMet, this value rose to 70% (*p* < 0.01).

## 4. Discussion

IRI is at the forefront of medical interest due to its ubiquitous presence in the operating room and the additional risk it poses. This has led to experimentation with numerous substances that seek to mitigate the harm caused. Non-pharmacological examples are ischemic preconditioning [12], which decreases the sensitivity of the organ to ischemia damage, and reperfusion to a lesser extent. We have focused our attention on SAMet, which, from a physiological and pathophysiological point of view, points to great potential for preventing liver damage in these circumstances and may therefore be a promising candidate to be incorporated into the clinic with this indication.

Analyzing the proposed parameters to see the impact of SAMet on them, we observed a regeneration of the ATP pool in liver tissue measurements. Its restoration represents a hepatoprotective effect because it plays several key roles in the maintenance of cellular homeostasis. Studies carried out by some authors suggest that the lack of ATP has clear clinical implications, such as the progression of liver damage in the presence of comorbid factors. Research in murine and human models of non-alcoholic steatohepatitis has shown that in patients with pre-existing liver damage, ATP loss is greater in response to a particular stimulus (fructose overload in these cases) and the time to recovery of ATP stores is longer [13]. Thus, the ability of SAMet to enhance ATP recovery presents a clear mechanistic benefit, even in cases where there are several sources of liver damage.

GSH levels in liver tissue were also elevated after SAMet administration, thus helping to restore a key element in the protection against free radicals [14]. In fact, Lu et al. demonstrated that mice lacking methionine adenosyltransferase-1A (necessary for SAMet production) had very low levels of GSH in liver tissue and developed steatohepatitis within 8 months [15]. The importance of GSH has been highlighted in different forms of liver damage, such as chronic cholestasis or alcohol-induced damage, because in all of them, one of the pathophysiological mechanisms of damage is the presence of oxidative stress, which can be reduced in the presence of GSH. Some authors such as Schauer et al. [16] have also carried out studies in IRI models in rats but administered GSH at doses of 100 μmol/h/kg, observing a reduction in mortality and in indirect markers of hepatocyte apoptosis. This study had a smaller sample size than ours, but it helps to identify a possible mechanism, the increase in GSH levels, by which SAMet has the beneficial effects that we have observed.

The caspase family is made up of highly conserved cysteine proteases that play an essential role in apoptosis. Mammalian caspases can be subdivided into three functional groups: initiator caspases (including caspase-9), executioner caspases (including caspase-3), and inflammatory caspases (including caspase-1). Initiator caspases initiate the apoptosis signal, while the executioner caspases carry out the mass proteolysis that leads to apoptosis. Inflammatory caspases do not function in apoptosis but are instead involved in inflammatory cytokine signaling and other types of cell death, such as pyroptosis.

Initially synthesized as inactive pro-caspases, caspases become rapidly cleaved and activated in response to granzyme B, death receptors, and apoptosome stimuli. Caspases will then cleave a range of substrates, including downstream caspases, nuclear proteins, plasma membrane proteins, and mitochondrial proteins, ultimately leading to cell death. In our study, and regarding caspase-1, -3, and -9, SAMet treatment was able to prevent the increase in these apoptosis-related markers produced in our IRI model. Interestingly, in the SAMet-treated animals, cleaved caspase-3 resulted in significantly lower values. Although both cleaved caspase-1 and -9 were not measured, which is a limitation of our study, the fact that the cleaved levels of the executioner caspase-3 were reduced in SAM-et-treated animals points to a reduction in apoptosis. Similar effects have been described in warm bile duct IRI, where suppression of apoptosis, as well as of oxidative stress and inflammatory reactions, were observed after orthotopic autologous liver transplantation [17].

In the case of cytokines, SAMet has been shown to decrease LPS-mediated TNF-α release in macrophages. A similar reduction was observed in our experimental model. It also increases the production of IL-10 which acts as an anti-inflammatory cytokine. Other studies performed in murine cell lines point to a possible increase in IL-10 synthesis by monocytes previously stimulated by endotoxin. Our results, while demonstrating an increase in IL-10 levels in the portal vein, showed a decrease in the vena cava. Although we have not found studies that specifically examine this phenomenon, some authors [18] have addressed the issue of cytokine level measurements in different body compartments and attribute this possible difference to the effect of the liver in capturing and processing these substances, as well as the possibility that in an inflammatory environment and damage, the levels of these substances are captured more avidly by the cells.

Another finding of interest of SAMet treatment in our model is related to the effect on IL-6, which is reduced at the level of the portal and cava veins after ischemia and subsequent reperfusion. However, hepatic IL-6 levels were not reduced. One possible explanation for this is the role of IL-6 in liver regeneration. Despite IL-6 being an acute phase reactant, other authors have investigated the impact of eliminating IL-6 production after acute liver injury, observing delayed tissue healing and more adverse tissue remodeling. In fact, Song et al. [19] conducted a study with SAMet, after the exposure of murine Kupffer cells to LPS, and observed an increase in IL-6 production, which was beneficial to the tissue. Plasma levels, which on the other hand were reduced in our study, have a role more related to a systemic inflammatory response and, therefore, at the liver level, would not be beneficial, as would be the case with a local increase in this organ. The observed increased levels of hepatic IL-6, when compared to the measurements in the porta and cava veins, are consistent with previous findings and current knowledge in immunology, since most IL-6 is produced by the migration to the liver of cells in response to the release of DAMPS as well as different chemotactic factors [20], but also, there is a substantial production by tissue-resident cells such as fibroblasts, resident immune cells, Kupffer cells and hepatocytes, which have been shown to produce IL-6 cytokine in large amounts when challenged by bacterial byproducts (such as LPS, as shown in our study) as well as by HGF [21]. The role of circulating cells in IL-6 levels in this scenario seems less substantial since experimental studies in rats have postulated that most of it is produced at the site of inflammation, showing that it is its concurrent production with other inflammatory mediators, namely IL-1β, that produces a febrile or systemic inflammatory response in the organism [22], and we found decreased levels of the latter with SAMet administration in our study. Nonetheless, it is a fact that as the inflammatory response progresses, there are other cells that adapt to release more inflammatory mediators, IL-6 in this case, such as endothelial cells [23] or circulating immune cells, but since this is a subsequent response, it would support our findings that reducing inflammation at the original site of injury reduces proinflammatory cytokine production subsequently.

Likewise, we have observed that SAMet produces a decrease in the levels of other key cytokines, such as IL-1β. This cytokine plays a crucial role in the inflammatory response, as mentioned above, and is partly responsible for the liver damage produced in acute and chronic injury. In a study by Yu et al. [24], an increase in its production was observed after SAMet administration, leading the authors to postulate that it could exert an endogenous inflammatory effect. However, the results are preliminary and were obtained from a metabolomic profile of macrophages, not being transferred to in vivo models.

The elevated level of LPO that we detected in both the plasma and liver tissue of the groups subjected to IRI suggests that the injury could be due, at least in part, to oxidative damage [25]. The prevention of this increase by treatment with SAMet leads us to think about the inhibition of this mechanism of LPO. Since GSH levels in the SAMet-treated groups were increased with respect to the non-treated groups, this inhibitory effect could be secondary, in part, to a higher rate of neutralization of oxygen free radicals, although we cannot rule out a direct effect on membrane lipids, protecting them against peroxidation.

As for bacterial translocation, we know that this is produced, among other mechanisms, by an increase in vascular permeability subsequent to the release of proinflammatory cytokines, protein kinases, reactive oxygen species or vasodilator compounds (such as NO). A systemic stress response may facilitate bacterial translocation in tissues peripheral to the damaged area, especially in particularly susceptible organs such as the intestine [26]. Endotoxin is a well-established marker of such a process, and a good alternative to others, including the measurement of bacterial DNA or peptidoglycans. Bacterial products such as this endotoxin are transported into the blood through unstructured tight junctions, and even through enterocytes, and bind to receptors (TRL4/CD14/MD-2) present on macrophages, lymphocytes and other cells to induce inflammation. In our study, we have observed a decrease in endotoxin levels at the level of the vena cava and portal vein after SAMet administration. In the past, other authors have studied the pernicious effects of bacterial translocation, which is markedly present in patients with chronic liver diseases, contributing to the damage caused by many of them, by determining endotoxin or other surrogate markers, looking for therapeutic options that would help to reduce them. Attempts have been made, including the administration of lactulose or broad-spectrum antibiotics, but no studies with SAMet for this indication have been performed. SAMet reduces this translocation through the effect of its signaling profile on the innate immune response and its role in the homeostasis of the organism, among other mechanisms. One example is the effect of a low SAMet concentration on immune response promoters, preventing the accumulation of H3K4me3, an epigenetic modification to DNA storage histone H3, in the promoters, which hinders the innate immune response [24].

Finally, it has been proposed that both the alteration of intracellular calcium homeostasis and the activation of phospholipase A2 are fundamental pathophysiological processes in the lesion caused by ischemia [27,28]. It is known that anoxia of solid organs determines a rapid fall of the normal calcium gradient across the cell membrane, resulting in an increase in intracellular calcium, with the consequent activation of phospholipase A2. In our work, we observed that IRI induced a significant decrease in phosphatidylcholine. This suggests a probable increase in phospholipase A2 activity. The effect was attenuated by SAMet, thus outlining another possible protective mechanism of this product, given the well-known role of phosphatidylcholine in the maintenance of cell membrane structure and function.

## 5. Conclusions

Regarding the status of SAMet in the therapeutic arsenal, to date, it has been tested in humans for several indications and is generally considered a fairly safe drug, except for some precautions in pregnant women and immunosuppressed patients. Its administration has been tested both intravenously (as in our study) and orally. Although the oral form has clear advantages in terms of its potential for use, there are far fewer trials, and therefore, it is not clear that it shares the benefits of intravenous administration.

Currently, many of these uses of SAMet are related to liver disease [6], with approved indications being hepatic cholestasis, intoxication by drugs that decrease GSH content (e.g., acetaminophen) and as an adjuvant in depression. For these situations, it has been demonstrated, for example in the case of hepatic cholestasis, by multiple randomized, double-blind, placebo-controlled clinical trials, that daily doses of SAMet of 800–1600 mg/day reduce biochemical indices of cholestasis and symptoms such as pruritus, even in pregnant patients. Its use in depression has so far been the subject of more than 11 clinical trials with more than 1000 patients [12] and has been shown to reduce the time required to obtain the desired effect with tricyclic antidepressants and some serotonin reuptake inhibitors. As for its usefulness in paracetamol intoxication, especially in comparison with N-acetylcysteine, this is more doubtful, and most of the existing evidence derives from experimental studies in animals, but it has nevertheless been incorporated into clinical practice. Its impact on alcoholic steatohepatitis is also interesting. In fact, a Spanish clinical trial conducted by Mato et al., with 123 patients receiving 1.2 g/day of oral SAMet, showed a 14% decrease in mortality in these patients [6]. All these studies have helped to demonstrate the efficacy and safety of SAMet administration in patients, but despite all of them, we still have very little evidence of its use in such a frequent problem as hepatic IRI.

The results obtained in the present study showed the efficacy of SAMet in reducing IRI injury, as we observed that it is beneficial, and therapeutically useful, in this experimental model of liver damage, thanks to its pleiotropic effects, some of which have been seen here, such as its ability to reduce bacterial translocation and the inflammatory response, or to replenish cellular ATP and GSH stores. These results seem to be promising for the potential use of SAMet in the clinic and support moving research on its effect on IRI into routine clinical practice, establishing its position as a future pharmacological treatment. However, this should be made with caution, since studies have indeed found differences in rat response to certain stimuli and immune responses as compared to humans. One example, and of relevance to the present study, would be the absence of tissue-resident macrophages in certain organs such as the lung [29], which would be important sources of cytokine production during systemic inflammation. Nonetheless rodents, and particularly rat models, have been widely used in IRI research, and although there is variation in the results depending on many technical aspects of the experiment (e.g., duration and level of vascular occlusion), the findings are useful to contribute to the understanding of the pathophysiology during this process in other models, including humans [30,31].

## Data Availability

The data that support the findings of this study are available from the corresponding author upon reasonable request.
